# Genetic Variants in the Hedgehog Interacting Protein Gene Are Associated with the FEV1/FVC Ratio in Southern Han Chinese Subjects with Chronic Obstructive Pulmonary Disease

**DOI:** 10.1155/2017/2756726

**Published:** 2017-08-27

**Authors:** Zili Zhang, Jian Wang, Zeguang Zheng, Xindong Chen, Xiansheng Zeng, Yi Zhang, Defu Li, Jiaze Shu, Kai Yang, Ning Lai, Lian Dong, Wenju Lu

**Affiliations:** ^1^State Key Laboratory of Respiratory Diseases, Guangzhou Institute of Respiratory Disease, The First Affiliated Hospital, Guangzhou Medical University, Guangzhou, Guangdong, China; ^2^Department of Pulmonary and Critical Care Medicine, The Johns Hopkins University School of Medicine, Baltimore, MD, USA; ^3^Department of Respiratory Medicine, Lufeng People's Hospital, Lufeng, Guangdong, China; ^4^Department of Respiratory Medicine, Xiangyang Central Hospital, Xiangyang, Hubei, China; ^5^Department of Respiratory Medicine, The Second Affiliated Hospital, Guangzhou Medical University, Guangzhou, Guangdong, China; ^6^Department of Laboratory Medicine, The First Affiliated Hospital, Guangzhou Medical University, Guangzhou, Guangdong, China

## Abstract

**Background:**

Convincing evidences have demonstrated the associations between* HHIP* and* FAM13a* polymorphisms and COPD in non-Asian populations. Here genetic variants in* HHIP* and* FAM13a* were investigated in Southern Han Chinese COPD.

**Methods:**

A case-control study was conducted, including 989 cases and 999 controls. The associations between SNPs genotypes and COPD were performed by a logistic regression model; for SNPs and COPD-related phenotypes such as lung function, COPD severity, pack-year of smoking, and smoking status, a linear regression model was employed. Effects of risk alleles, genotypes, and haplotypes of the 3 significant SNPs in the* HHIP* gene on FEV_1_/FVC were also assessed in a linear regression model in COPD.

**Results:**

The mean FEV_1_/FVC% value was 46.8 in combined COPD population. None of the 8 selected SNPs apparently related to COPD susceptibility. However, three SNPs (rs12509311, rs13118928, and rs182859) in* HHIP* were associated significantly with the FEV_1_/FVC% (*P*_max_ = 4.1 × 10^−4^) in COPD adjusting for gender, age, and smoking pack-years. Moreover, statistical significance between risk alleles and the FEV_1_/FVC% (*P* = 2.3 × 10^−4^), risk genotypes, and the FEV_1_/FVC% (*P* = 3.5 × 10^−4^) was also observed in COPD.

**Conclusions:**

Genetic variants in* HHIP* were related with FEV_1_/FVC in COPD. Significant relationships between risk alleles and risk genotypes and FEV_1_/FVC in COPD were also identified.

## 1. Introduction

Chronic Obstructive Pulmonary Disease (COPD) is a complex disease characterized by airflow limitation that is not completely reversible. It is projected to rise to rank fifth in disease burden by 2020 around the world [1], and in USA, it ranks as the third leading cause of death [2]. Although tobacco smoking was suggested to be the major adverse factor for the progress of COPD, about 85% of the smokers did not develop into clinically relevant airflow obstruction [3], implying that genetic susceptibility might play a crucial role in the development of COPD. However, these genetic factors are not yet fully understood. Few susceptibility genes other than*α*_1_-*ANTITRYPSIN* have been convincingly identified yet [4].

Genome-wide association studies (GWAS), which have revolutionized the identification of susceptibility genes for polygenic diseases, figured out statistically significant relations regarding the* FAM13a* and* HHIP* and COPD in non-Asian populations [5–8]. Evidences identified that* FAM13a* and* HHIP* might be involved in the etiology of COPD [9, 10]. For instance, several GWAS studies have showed the significant associations between the* HHIP* loci and COPD susceptibility [9, 11]. In view of the great differences in genetic profiles of different ethnicity, replication works in other populations and with more SNPs are warranted. Therefore, the genetic relationships between the* FAM13a* and* HHIP* genes and COPD were conducted by the Southern Han Chinese COPD case-control study. We wanted to explore whether a selection of SNPs in* FAM13a* and* HHIP* was related to COPD and COPD-related phenotypes.

## 2. Methods and Materials

### 2.1. Study Population

The study design and subject recruitment have been described previously [12]. In short, all participants were genetically unrelated ethnic Southern Han Chinese and from Guangdong and Hubei Province, 40 to 80 years old. All 989 hospitalized COPD patients manifested after bronchodilator the FEV_1_/FVC% values of <0.7 and were successively recruited from September 2010 to September 2013 in the Department of Respiratory Medicine (an ongoing project). “Study Ι” involved 594 cases and 600 controls from the First Affiliated Hospital of Guangzhou Medical University (Guangzhou, Guangdong, China). “Study II” was conducted on participants (395 cases and 399 controls) derived from Xiangyang, Central Hospital (Xiangyang, Hubei, China), to verify the results from the Study Ι population. The 999 control subjects demonstrated normal lung function (postbronchodilator FEV_1_ > 80% predicted; the FEV_1_/FVC% > 0.7) and were randomly recruited from the Health Examination Center of the same hospital during the same time period when patients were recruited to maximally match the situation of the regarding cases; efforts were made to frequency-match COPD by age (±5 years) and sex. Participants were excluded with regard to concomitant respiratory disorders, lung surgery, and pregnancy, and so forth before enrollment.

### 2.2. Data Collection

Prebronchodilator and postbronchodilator spirometry were conducted on subjects according to standardized protocol with the EasyOne Spirometer (NDD, Inc., Andover, MA) [13]. Post-bronchodilator spirometry was measured probably 20 minutes after performing 180 *μ*g of albuterol via metered dose inhaler. Few patients could not finish at least three complete spirometries or their values of measurements mildly exceeded spirometric criteria; the data of those patients included were determined by the investigators' discretion. Predicted FEV1% and FEV1/FVC% were evaluated using the Global Lung Function Initiative (GLI) equations [14]. Airway obstruction was diagnosed by the GLI definition of FEV1/FVC < the Lower Limit of Normal (LLN) and *z*-score. Data was described by the mean ± SD (strictly 1.96 *z*-scores), which extended from the 2.5th to the 97.5th centile of the distribution (*z*-scores indicate how many standard deviations a measurement is from its predicted value). Categorization of the severity of airway obstruction was made using the four-category scale (mild: *z* ≥ −2; moderate: −2 > *z* ≥ −2.5; moderately severe: −2.5 > *z* ≥ −3; severe: −3 > *z* ≥ −4, −4 > *z*) [15]. Prior to formal recruitment, informed consent was signed by each of the qualified subjects as written form, and structured questionnaires were conducted by professional staff to collect information on demographic data and environmental exposure history such as tobacco cigarette smoke and so forth. Subjects were subgrouped as smokers, ex-smokers, and nonsmokers. The definitions of nonsmokers, ex-smokers, nonsmokers, and pack-years of smoking were detailed in our earlier study [16]. After the interview, a ~5 ml venous blood sample was collected from each participant. This study was approved by each participating center's Institutional Ethical Committee and was administrated by the principles of the Declaration of Helsinki (Ethics Committee of the First Affiliated Hospital: GZMC 2010-03-28; Xiangyang Central Hospital of Hubei: 2011-01-23).

### 2.3. Polymorphisms Selection and Genotyping Assays

Based on the findings from the GWAS of COPD in non-Asian populations, three top SNPs (rs1903003, rs2869967, and rs7671167) in* FAM13a* gene and five top SNPs (rs12504628, rs12509311, rs13118928, rs1512281, and rs1828591) in* HHIP* gene, which were found to be significantly associated with COPD risk, were selected [5–8] (the top SNP defined as *P* value was the most significant). Because we chose these SNPs based on the reported top findings of COPD GWAS in non-Asian populations, rather than the database such as dbSNP and Hapmap, we just did the replication analyses in the present study without considering the linkage disequilibrium (*LD*) (*D*′ ≥ 0.9864, *r*^2^ ≥ 0.9621 between the five SNPs in* HHIP* in controls) (see Table S1 in Supplementary Material available online at https://doi.org/10.1155/2017/2756726).

QIAGEN Blood DNA Kit was employed to extract the genomic DNA of each participant. ABI PRISM 7500 Sequence Detection System (Applied Biosystems, Foster City, CA) with allelic discrimination method was chosen for genotyping [17]. Primers and probes assay as well as polymerase chain reaction has been described sufficiently in our earlier study [16]. The genotypes were automatically calculated by Sequence Detection Systems software 2.3 (Applied BioSystems). The accordance rate of each SNP was 100% for the duplicates of 10% of samples.

### 2.4. Statistical Analysis

Baseline characteristics were analyzed for quantitative traits using *t*-tests and for binary traits using a two-sided *χ*^2^ test. Goodness of fit to the Hardy-Weinberg equilibrium expectation in controls was also assessed by the *χ*^2^ for each SNP. The associations between COPD susceptibility and SNP genotypes were calculated by an unconditional logistic regression model after adjustment for gender, age, and smoking pack-years under different genetic models. The associations between COPD-related phenotypes and the three significant SNPs were tested by a linear regression model after adjusting for gender, age, and smoking pack-years with an additive genetic model. Effects of risk alleles and genotypes of the 3 significant SNPs on the FEV1/FVC% were also calculated using a linear regression model adjusting for gender, age, and smoking pack-years. To control the family-wise type I error rate at a 0.05 level, a Bonferroni correction was applied. With 8 SNPs between comparisons, each individual 2-sided test was considered statistically significant relative to a 0.006 significance level. Spirometry predicted values and *z*-scores were derived for each subject in each dataset using prediction equations from GLI-2012 [14] using specially developed GLI-Excel-Calculator in the supplementary file from the Quanjer et al. study [15]. Statistical analyses were all evaluated using the SAS9.2 software and statistical power measured by Quanto 1.2.

## 3. Results

### 3.1. Characteristics of the Study Population

The characteristics of the combined 989 COPD patients and 999 controls were described in Table 1. Briefly, the COPD cases and controls appeared to be age matched (*P* = 0.807). As expected, COPD cases indicated a more serious condition on pulmonary function compared to controls. The pack-year of cigarette smoking and the frequency of male in COPD were greater than that in control subjects. The ex-smokers were more likely suffering from COPD, while more nonsmokers or smokers were to be control subjects. Among the 989 COPD cases, 155 (15.7%) were defined as mild COPD, 156 (15.8%) as moderate, 368 (37.2%) as severe, and 310 (31.3%) as very severe. The spearman coefficients between the FEV1/FVC% and FEV1 were 0.5526 and −0.0718 in subjects with COPD and controls, respectively (Table S2). Moreover, Study I and Study II revealed almost identical change tendency as the combined population (principal components analysis for heterogeneity test between the two groups, *P* = 0.226, Table S3).

### 3.2. Genetic Association

Case-control analyses exploring further associations identified that none of the SNPs was significantly related to COPD risks without adjustment. Linear regression analyses showed that three SNPs (rs12509311, rs13118928, and rs1828591) in the* HHIP* gene were significantly associated with the FEV1/FVC% in COPD with adjustment in combined population (*P*_max_ = 4.1 × 10^−4^) (Table 2). Therefore, additional models with adjusting for gender, age, and pack-years of smoking were conducted for further analyses. Similarly, none of the SNPs were figured out to be genetic associations with COPD susceptibility (Table 3). However, rs12509311, rs13118928, and rs1828591 were only significantly associated with the FEV1/FVC% in COPD (*P* = 4.1 × 10^−4^, 2.8 × 10^−4^, and 4.1 × 10^−4^, resp.) (Table 4) rather than in the controls (Table S4). Risk alleles and genotypes of the three SNPs analyses revealed that there were significant associations between risk alleles and the FEV1/FVC% (*P* = 2.3 × 10^−4^) as well as between risk genotypes and the FEV1/FVC% (*P* = 3.5 × 10^−4^) in subjects with COPD after adjusting for gender, age, and pack-years of smoking (Table 5).

## 4. Discussion

To date, COPD remains a major worldwide and increasing health problem incurred by multiple genetic and environmental factors [9]. GWAS have identified several susceptibility genes for COPD, including* FAM13a*,* HHIP*,* CHRNA3/CHRNA5/IREB2*,* RIN3*,* MMP3/MMP12*, and* TGFB2* [7, 8, 10, 11, 18–23]. These loci identified in GWAS were conducted not only in population of European and African descent, but also in Asians [24–26]. For* FAM13a*, the susceptibility for COPD was also found in Hispanics [27]. Recently, studies have revealed the associations between* HHIP* and lung function [24, 28]. Kim et al. found that, in the KOLD cohort study, two SNPs (rs11938704 and rs10013495) near* HHIP* were significantly associated with FEV1 (*P* = 0.0001 and 0.001, resp.) in COPD [28]. However, evidences from those studies have not yet been reached for the other COPD phenotypes. In the current case-control study, we explored the role of multiple variants of* FAM13a* and* HHIP* and assessed their relationships not only with COPD, but also with COPD-related phenotypes in above described Southern Chinese Han population. In this study, we analyzed 3 top SNPs in* FAM13a* gene and 5 top SNPs in* HHIP* gene, which previously showed statistical significance based on GWAS, but the genetic associations with COPD susceptibility were not identified in the current study. Genetic variants in* HHIP* were associated with the FEV1/FVC% in COPD cases with adjustment for gender, age, and pack-years of smoking. In addition, we also identified significant relationships between risk alleles or risk genotypes and the FEV1/FVC% in COPD. These results indicated that the* HHIP* gene might contribute to the variation of the FEV1/FVC%.

It is biologically possible that* HHIP* may be involved in the etiology of COPD. The gene of* HHIP* encodes a membrane glycoprotein, which is an endogenous antagonist for the protein of Sonic Hedgehog (SHH) [29, 30]. The evolutionarily highly conserved hedgehog signaling pathway is functionally implicated in a variety of physiological or pathological processes, including lung organogenesis, embryogenesis, chronic inflammation, and carcinogenesis, as well as for response of the airway epithelium exposed to smoking [31–33]. Hedgehog signaling by* HHIP* is playing crucial roles in lung morphogenesis, especially in the stage during early lung branching. Knockout of* HHIP* in mice led to the inhibition of lung bud branching and neonatal respiratory failure. Changes of* HHIP* protein or its expression in humans might change the development of lung or repair mechanisms [34, 35]. Earlier GWAS studies demonstrated significant associations between the* HHIP* loci and COPD risk [18, 21]. The associations of* HHIP* with COPD risk were also shown in the Rotterdam study and the Polish cohort and one study among Southwestern Chinese Han population [21, 36]. However, the association between* HHIP* and COPD risk was not identified in the Southern Chinese Han population in this study. The explanations for these conflicting results might be as follows: first, ethnic differences contributed to this variability [37, 38]. For instance, study identified novel association between certain genes with chronic diseases in Hispanic population, but in non-Hispanic the significance was rather limited, including the two novel loci (rs858249 and rs286499) in or near the genes* KLHL7/NUPL2* and* DLG2* [27]. Similarly, associations identified in European-ancestry population might be less significant in non-European populations due to difference in ethnicity [39]. Second, the reason might be due to the different consumption of cigarettes. In the previously mentioned Rotterdam study, SNPs in* HHIP* were more associated with COPD in the subgroup of heavy smoker, and genetic variation near the* HHIP* gene was significantly associated with risk of COPD depending on the quantity of pack-years of smoking. However, the relatively mild smoking levels of the population in our study might result in the nonsignificant association between SNPs in* HHIP* and COPD susceptibility (Table S5). Third, the reasons could be due to the differences in COPD severity. In the Polish cohort, SNPs in the 4q31 chromosome region were greatly associated with severe COPD [36] and the* HHIP* SNP rs10519717 was associated with the severity of COPD in the Southwestern Chinese Han population. Fourth, the reasons could be differences determined by characteristics of population such as age, gender distribution, and height. For instance, earlier GWAS found out that genetic variants in* HHIP* were significantly related to adult height in European and Korean populations [21, 40]. Fifth, differences resulted from measurements of lung function. In most of the constituent studies, postbronchodilator spirometry was not measured. However, previous study reported that substantial misclassification would occur by using prebronchodilator spirometry to diagnose COPD in mild COPD case subjects [41]. In the present study, postbronchodilator spirometry was measured and mild COPD cases were included. Finally, the discrepancies might come from different pathologic stages of airflow obstruction. Postbronchodilator spirometry was applied for the detection of airflow obstruction, which was regarded as the formal diagnosis of COPD. Patients with partly or fully reversible airflow obstruction might have fundamentally different pathological development contributing to airflow obstruction. The underlying bias was that there might be some patients with misclassification of either an asthma diagnosis or the COPD diagnosis. Both asthma and COPD are common diagnoses and could coexist in the same cases. If the SNP effects were specific to asthma and not present in COPD, the contribution of the SNPs to COPD would be overestimated if including the patients with asthma. However, we investigated the effects with exclusion of patients with known asthma from the case subjects in a subset of the data with asthma diagnosis available.

Furthermore, many previous studies have demonstrated significant genetic relationships between* HHIP* loci and the FEV1/FVC% such as in the GWAS of the Framingham Heart Study population [7], the Cohorts for Heart and Aging Research in Genomic Epidemiology and SpiroMeta consortia [5, 42], Evaluation of COPD Longitudinally to Identify Predictive Surrogate Endpoints (ECLIPSE), and International COPD Genetics Network (ICGN) subjects [43]. The FEV1/FVC% is an important quantitative characteristic of COPD. The decreased FEV1/FVC%, an indicator of airflow obstruction that is independent of lung size, is the important criterion for defining an obstructive ventilator defect [44]. We also identified a significant association between SNPs in* HHIP* and the FEV1/FVC% in COPD in Southern Chinese Han population. The results showed that rs12509311, rs13118928, and rs1828591 were associated with the FEV1/FVC% among cases only. While some other studies did not draw such a conclusion [24, 28], potential explanations might be the differences in genetic heterogeneity, statistical power, and population characteristic. Generally, genetic heterogeneity would be an important factor to explain this conflict. Even if the same genetic variant is involved in each population, the *LD* relationships of this variant with neighboring genetic polymorphisms might also vary between ethnic groups. Moreover, our statistical powers were all ≥95% to support our claim. The FEV1/FVC% is also determined by population characteristics including height and heritability. Given that COPD airflow obstruction is typically determined by the FEV1/FVC%, the current study suggested that* HHIP* was associated with COPD airflow obstruction. Besides, the relationship between SNPs in* HHIP* and the other COPD phenotypes were also observed, but significant associations were not demonstrated, including FEV_1_, COPD severity, and smoking status. Except the above-mentioned three explanations (genetic heterogeneity, statistical power, and population characteristic), differences in smoking exposure, current smoking status, entry criteria, and geographic origin of the population might also make a contribution to phenotypic heterogeneity and lead to the discrepancies. Additionally, significant relationships between risk alleles or risk genotypes and the FEV1/FVC% in COPD also were demonstrated in our study. Explanations on associations between SNPs in* HHIP* and the FEV1/FVC% could be also applicable here because of risk alleles or risk genotypes actually resulting from the combination of number of the three risk SNPs. For further study, functional experiments should be needed.

In conclusion, genetic variants in* HHIP* were found to be associated with the FEV1/FVC% in COPD cases. However, the relationship between* HHIP* and* FAM13a* polymorphisms and COPD susceptibility was not identified. Significant relationships between risk alleles and the FEV1/FVC% as well as risk genotypes and the FEV1/FVC% in subjects with COPD were identified. Given the uniform conclusions in Chinese populations, replication studies with more populations and more SNPs are required in the future study.

## Supplementary Material

Table S1: Linkage disequilibrium (D´ and r2) between SNPs in HHIP in controls.
Table S2: Spearman coefficient between lung function, age, pack-year, and COPD severity in COPD and controls.
Table S3: Principal components analysis for Heterogeneity test before combined Study I and Study II.
Table S4: Linear regression analyses of the genetic associations among SNPs and lung function, smoking amount in controls.
Table S5: Interaction effects between SNPs and pack-year or age.

## Figures and Tables

**Table 1:** Demographic characteristics in COPD patients and controls.

Variables	Study Ι			Study ΙΙ				Combined		
	Cases	Controls	*P* ^a^	Cases	Controls	*P* ^a^	*P* ^b^	Case	Control	*P* ^a^
	(*N* = 594)	(*N* = 600)		(*N* = 395)	(*N* = 399)			(*N* = 989)	(*N* = 999)	
	$\overset{-}{x}\;\;$± sd	$\overset{-}{x}\;\;$± sd		$\overset{-}{x}\;\;$± sd	$\overset{-}{x}\;\;$± sd			$\overset{-}{x}\;\;$± sd &	$\overset{-}{x}\;\;$± sd	
	*n* (%)	*n* (%)		*n* (%)	*n* (%)			*n* (%)	*n* (%)	
Age (years)	60.2 ± 9.9	60.6 ± 7.5	0.597	62.2 ± 9.5	61.3 ± 8.6	0.162	0.201	61.0 ± 9.7	60.9 ± 8.5	0.807
Gender, male (%)	516 (86.8)	497 (82.9)	0.186	339 (85.8)	208 (52.1)	<**0**.**001**	<**0**.**001**	855 (86.4)	705 (70.6)	<**0**.**001**
FEV1 (L)	0.98 ± 0.54	2.43 ± 0.56	<**0**.**001**	0.92 ± 0.37	2.47 ± 0.54	<**0**.**001**	0.051	0.96 ± 0.37	2.44 ± 0.55	<**0**.**001**
FEV1 predict pre-BD^*∗*^ (%)	42.2 ± 20.5	94.3 ± 15.7	<**0**.**001**	41.8 ± 18.3	95.7 ± 14.6	<**0**.**001**	0.221	42.1 ± 18.2	94.8 ± 15.3	<**0**.**001**
FEV1/FVC (%)	46.9 ± 17.7	81.4 ± 6.8	<**0**.**001**	46.7 ± 16.9	81.7 ± 6.4	<**0**.**001**	0.536	46.8 ± 16.9	81.5 ± 6.7	<**0**.**001**
Pack-years	42.1 ± 27.6	16.9 ± 13.3	<**0**.**001**	35.6 ± 20.9	10.8 ± 4.9	<**0**.**001**	**0**.**001**	39.4 ± 19.5	14.4 ± 8.6	<**0**.**001**
Smoking status			<**0**.**001**			<**0**.**001**	0.092			<**0**.**001**
Smoker	74 (12.5)	179 (29.9)		45 (11.4)	118 (9.6)			119 (12.0)	297 (29.7)	
Ex-smoker	421 (70.9)	6 (1.0)		282 (71.4)	3 (0.8)			703 (71.1)	9 (0.9)	
Nonsmoker	99 (16.6)	415 (69.1)		68 (17.2)	278 (9.6)			167 (16.9)	693 (69.4)	
GLI							0.479			
Mild	89 (15.0)			66 (16.7)				155 (15.7)		
Moderate	94 (15.8)			62 (15.7)				156 (15.8)		
Moderately severe	224 (37.7)			144 (36.5)				368 (37.2)		
Severe	187 (31.5)			123 (31.1)				310 (31.3)		

^a^ *P* values for a two-sided *χ*^2^ test or *t*-test; ^b^*P* values for the test of homogeneity by Breslow-Day test or *Q* test; FEV1: forced expiratory volume in 1 second; ^*∗*^pre-BD: prebronchodilator; FVC: forced vital capacity; in bold is *P* < 0.006.

**Table 2:** Genetic association results in the case-control study (left column) and associations with FEV1 and the FEV1/FVC% in the subjects with COPD (right column).

SNP	Gene	Chr	Location^a^	Minor	*P*_HWE_^b^	Logistic regression			Linear regression in COPD (crude)				Linear regression in COPD (adjusted)				
						MAF			FEV1		FEV1/FVC%		FEV1		FEV1/FVC%		
				Allele		COPD	Control	*P* ^c^	*β*	*P*	*β*	*P*	*β*	*P* ^c^	*β*	*P* ^c^	Power^d^
Combined population (number of COPD = 989, control = 999)																	
rs1903003	*FAM13A*	4	88965146	C	0.23	0.430	0.467	0.05	0.008	0.628	0.59	0.31	0.01	0.85	0.64	0.41	—
rs2869967	*FAM13A*	4	88948181	T	0.21	0.468	0.519	0.03	−0.01	0.415	0.48	0.39	−0.02	0.47	0.52	0.50	—
rs7671167	*FAM13A*	4	88962828	C	0.72	0.455	0.487	0.11	−0.44	0.597	0.35	0.55	−0.01	0.58	0.34	0.66	—
rs12504628	*HHIP*	4	144515172	C	0.56	0.242	0.262	0.37	2.33	0.017	**3**.**45**	**1**.**32e**^−**4**^	0.06	0.07	2.01	0.01	—
rs1512281	*HHIP*	4	144513749	G	0.50	0.244	0.264	0.36	2.28	0.018	**3**.**42**	**1**.**33e**^−**4**^	0.06	0.09	1.95	0.02	—
rs12509311	*HHIP*	4	144557510	T	0.99	0.229	0.251	0.27	2.66	0.007	**4**.**26**	**2**.**72e**^−**6**^	0.05	0.12	**2**.**68**	**4**.**1e**^−**4**^	0.95
rs13118928	*HHIP*	4	144565237	G	0.95	0.230	0.253	0.23	2.60	0.008	**4**.**19**	**3**.**87e**^−**6**^	0.06	0.09	**2**.**74**	**2**.**8e**^−**4**^	0.96
rs1828591	*HHIP*	4	144559628	G	0.99	0.229	0.251	0.27	2.66	0.007	**4**.**26**	**2**.**72e**^−**6**^	0.05	0.12	**2**.**68**	**4**.**1e**^−**4**^	0.95

^a^The version of chromosome location is GRCh38 in GenBank of PubMed Centre; ^b^*P*_HWE_: calculated by the Hardy-Weinberg equilibrium among the control subjects. ^c^Adjusted in a logistic or linear regression model that included gender, age, and pack-years of smoking. ^d^Statistical powers: measured by linear regression in the FEV1/FVC%. Minimum call rate of the alleles is 99.33%. In bold is *P* < 0.006.

**Table 3:** Analyses of associations between 3 SNPs in the *HHIP* gene and COPD.

Genotypes/alleles	Case	Control	Crude	Adjusted	
	*n* (%)	*n* (%)	OR (95% CI)	OR (95% CI)	*P* ^a^
Total number of subjects	989	999			
Total number of alleles	1978	1998			
*rs12509311C>T*					
Codominant model					
CC	581 (59.0)	559 (56.1)	1.00 (ref.)	1.00 (ref.)	
CT	356 (36.1)	374 (37.6)	0.92 (0.76–1.10)	0.85 (0.62–1.17)	0.32
TT	48 (4.9)	63 (6.3)	0.73 (0.50–1.09)	0.69 (0.38–1.27)	0.24
Additive model			0.89 (0.77–1.03)	0.84 (0.66–1.07)	0.16
Dominant model					
CT + TT	404 (41.0)	437 (43.9)	0.89 (0.74–1.06)	0.82 (0.61–1.11)	0.21
Recessive model					
CC + CT	937 (95.1)	933 (93.7)	1.00 (ref.)	1.00 (ref.)	
TT	48 (4.9)	63 (6.3)	0.76 (0.52–1.12)	0.74 (0.41–1.33)	0.31
*rs13118928A>G*					
Codominant model					
AA	581 (58.9)	559 (56.0)	1.00 (ref.)	1.00 (ref.)	
GA	358 (36.3)	374 (37.4)	0.92 (0.77–1.11)	0.85 (0.62–1.17)	0.33
GG	48 (4.8)	66 (6.6)	0.70 (0.47–1.03)	0.69 (0.38–1.25)	0.22
Additive model			0.88 (0.76–1.02)	0.84 (0.66–1.07)	0.15
Dominant model					
GA + GG	406 (41.1)	440 (44.0)	0.89 (0.74–1.06)	0.82 (0.61–1.11)	0.20
Recessive model					
AA + GA	939 (95.2)	933 (93.4)	1.00 (ref.)	1.00 (ref.)	
GG	48 (4.8)	66 (6.6)	0.72 (0.49–1.06)	0.73 (0.40–1.31)	0.29
*rs1828591A>G*					
Codominant model					
AA	581 (59.0)	559 (56.1)	1.00 (ref.)	1.00 (ref.)	
GA	356 (36.1)	374 (37.6)	0.92 (0.76–1.10)	0.85 (0.62–1.17)	0.32
GG	48 (4.9)	63 (6.3)	0.73 (0.50–1.09)	0.69 (0.38–1.27)	0.24
Additive model			0.89 (0.77–1.03)	0.84 (0.66–1.07)	0.16
Dominant model					
GA + GG	404 (41.0)	437 (43.9)	0.89 (0.74–1.06)	0.82 (0.61–1.11)	0.21
Recessive model					
AA + GA	937 (95.1)	933 (93.7)	1.00 (ref.)	1.00 (ref.)	
GG	48 (4.9)	63 (6.3)	0.76 (0.52–1.12)	0.74 (0.41–1.33)	0.31

^a^Adjusted in a logistic regression model that included gender, age, and pack-years of smoking.

**Table 4:** Linear regression analyses of the genetic associations among SNP and lung function, COPD severity, and smoking amount in the subjects with COPD.

	Subjects with COPD (*N* = 989)											
	FEV1		FEV1 pre-BD		The FEV1/FVC%		GOLD		Pack-years		Smoking status	
	*β* (SE)	*P* ^a^	*β* (SE)	*P* ^a^	*β* (SE)	*P* ^a^	*β* (SE)	*P* ^a^	*β* (SE)	*P* ^a^	*β* (SE)	*P* ^a^
rs12509311	0.052 (0.033)	0.122	0.041 (0.025)	0.105	**2**.**683** (**0**.**757**)	**4**.**1e**^−**4**^	−0.020 (0.044)	0.644	1.317 (1.257)	0.153	−0.030 (0.026)	0.251
rs13118928	0.057 (0.033)	0.089	0.040 (0.025)	0.108	**2**.**743** (**0**.**754**)	**2**.**8e**^−**4**^	−0.027 (0.044)	0.538	1.392 (1.257)	0.145	−0.036 (0.026)	0.172
rs1828591	0.052 (0.033)	0.122	0.041 (0.025)	0.105	**2**.**683** (**0**.**757**)	**4**.**1e**^−**4**^	−0.020 (0.044)	0.644	1.317 (1.257)	0.153	−0.030 (0.026)	0.251

^a^Calculated with adjusting gender, age, and pack-years of smoking in linear regression. Statistical powers are 0.85, 0.86, and 0.85 for rs12509311, rs13118928, and rs1828591 in the FEV1/FVC%, respectively. The data presented are *β* (SE) with two-sided *P* value. FEV1, forced expiratory volume in 1 s; FVC, forced vital capacity; in bold is *P* < 0.006.

**Table 5:** Effect of risk allele and genotypes of 3 SNPs in the *HHIP* gene on the FEV1/FVC% in the subjects with COPD.

	Subjects with COPD (*N* = 989)			
	Crude		Adjusted	
	*β* (SE)	*P*	*β* (SE)	*P* ^a^
Risk allele	1.328 (0.309)	**1**.**9e**^−**5**^	1.077 (0.292)	**2**.**3e**^−**4**^
Risk genotypes^b^	2.058 (0.490)	**2**.**7e**^−**5**^	1.651 (0.462)	**3**.**5e**^−**4**^

^a^Adjusted in a linear regression model that included gender, age, and pack-years of smoking; risk allele means number of the three SNPs in combination. ^b^Genotype combinations in *HHIP*: rs12509311T variant genotypes (CT or TT), rs13118928G variant genotypes (AG or GG), or rs1828591G variant genotypes (AG or GG) are defined as risk genotypes. Risk genotype means number of variant genotypes in combination. The data presented are *β* (SE) with two-sided *P* value. In bold is *P* < 0.006.

## Abbreviations

COPD: Chronic obstructive pulmonary disease
FEV_1_: Forced expiratory volume in 1 s
FVC: Forced vital capacity
*FAM13a*: Family with sequence similarity 13, member a
*HHIP*: Hedgehog interacting protein
GWAS: Genome-wide association study
SNP: Single-nucleotide polymorphism.
