# Supplementary material for: Genetic Variants in the Hedgehog Interacting Protein Gene Are Associated with the FEV1/FVC Ratio in Southern Han Chinese Subjects with Chronic Obstructive Pulmonary Disease
Source: Biomed Res Int. 2017 Aug 27;2017:2756726. doi: 10.1155/2017/2756726 (PMC5591965; doi:10.1155/2017/2756726)
Supplement: Supplementary file 1 — Table S1: Linkage disequilibrium (D´ and r2) between SNPs in HHIP in controls. Table S2: Spearman coefficient between lung function, age, pack-year, and COPD severity in COPD and controls. Table S3: Principal components analysis for Heterogeneity test before combined Study I and Study II. Table S4: Linear regression analyses of the genetic associations among SNPs and lung function, smoking amount in controls. Table S5: Interaction effects between SNPs and pack-year or age. [file 2756726.f1.docx]

**Table S1.** Linkage disequilibrium (D´ and r*^2)^* between SNPs in *HHIP* in controls

| **SNP** | rs12509311 | rs13118928 | rs1828591 | rs12504628 | rs1512281 |
| --- | --- | --- | --- | --- | --- |
| rs12509311 | — | 1.0000 | 1.0000 | 0.9737 | 0.9621 |
| rs13118928 | 1.0000 | — | 1.0000 | 0.9737 | 0.9621 |
| rs1828591 | 1.0000 | 1.0000 | — | 0.9737 | 0.9621 |
| rs12504628 | 0.9919 | 0.9919 | 0.9919 | — | 0.9883 |
| rs1512281 | 0.9864 | 0.9864 | 0.9864 | 0.9947 | — |

*r^2^* values are given above the diagonal; *D*’ values are given below the diagonal.

**Table S2.** Spearman coefficient between lung function, age, pack-year, and COPD severity in COPD and controls.

|  | FEV1 | FEV1/FVC | FEV1_pre_BD | Age | Pack-year | GOLD |
| --- | --- | --- | --- | --- | --- | --- |
| FEV1 | — | 0.5526 | 0.1895 | -0.0022 | 0.0033 | **-0.7127** |
| FEV1/FVC | -0.0718 | — | **-0.2511** | **0.1894** | -0.0738 | **-0.7117** |
| FEV1_pre_BD | **0.6730** | **-0.2305** | — | **-0.5865** | **0.1655** | **0.2909** |
| Age | -0.0311 | **-0.1876** | -0.0380 | — | **-0.0999** | **-0.2326** |
| Pack-year | **0.3510** | **-0.1463** | **0.3707** | **0.4071** | — | **0.1584** |
| GOLD | — | — | — | — | — | — |

COPD are given above the diagonal; Control are given below the diagonal.

In Bold is *P* < 0.05.

**Table S3.** Principal components analysis for Heterogeneity test before combined Study I and Study II.

|  | Step1 | | | |  | Step2 | |  | Step3 |  | Step4 | |
| --- | --- | --- | --- | --- | --- | --- | --- | --- | --- | --- | --- | --- |
| Study | Principal Components | | | |  | Select Component= RΙ-1, RΙΙ-1 | |  | Logit regression ^a^ |  | Heterogeneity | |
|  | Component | Eigenvalue | Proportion | Cumulative |  | Variable | Eigenvectors |  | β ( 95%CI) |  | Q ^b^ | *P* |
| Replication Ι |  |  |  |  |  |  |  |  | -5.34(-6.24,-4.45) |  |  |  |
|  | RΙ-1 | 3.73646 | 0.4671 | 0.4671 |  | Age | 0.05372 |  |  |  |  |  |
|  | RΙ-2 | 1.25128 | 0.1564 | 0.6235 | . | Gender | 0.00899 |  |  |  |  |  |
|  | RΙ-3 | 0.95769 | 0.1197 | 0.7432 |  | FEV1 | 0.48319 |  |  |  |  |  |
|  | RΙ-4 | 0.85212 | 0.1065 | 0.8497 |  | FEV1_pre_BD | 0.47122 |  |  |  |  |  |
|  | RΙ-5 | 0.64315 | 0.0804 | 0.9301 |  | FEV1/FVC | 0.43708 |  |  |  |  |  |
|  | RΙ-6 | 0.3264 | 0.0408 | 0.9709 |  | Pack-year | -0.28326 |  |  |  |  |  |
|  | RΙ-7 | 0.19824 | 0.0248 | 0.9957 |  | Smoking Status | 0.42759 |  |  |  |  |  |
|  | RΙ-8 | 0.03467 | 0.0043 | 1 |  | GOLD | 0.29565 |  |  |  |  |  |
| Replication ΙΙ |  |  |  |  |  |  |  |  | -4.56(-5.46,-3.65) |  |  |  |
|  | RΙΙ-1 | 4.4073 | 0.5509 | 0.5509 |  | Age | -0.40346 |  |  |  |  |  |
|  | RΙΙ-2 | 1.50146 | 0.1877 | 0.7386 |  | Gender | 0.24399 |  |  |  |  |  |
|  | RΙΙ-3 | 0.72139 | 0.0902 | 0.8288 |  | FEV1 | 0.40999 |  |  |  |  |  |
|  | RΙΙ-4 | 0.51215 | 0.064 | 0.8928 |  | FEV1_pre_BD | 0.4292 |  |  |  |  |  |
|  | RΙΙ-5 | 0.42504 | 0.0531 | 0.9459 |  | FEV1/FVC | 0.41304 |  |  |  |  |  |
|  | RΙΙ-6 | 0.23259 | 0.0291 | 0.975 |  | Pack-year | -0.33871 |  |  |  |  |  |
|  | RΙΙ-7 | 0.17352 | 0.0217 | 0.9967 |  | Smoking Status | -0.17541 |  |  |  |  |  |
|  | RΙΙ-8 | 0.02654 | 0.0033 | 1 |  | GOLD | 0.33058 |  |  |  |  |  |
| Pooled effect |  |  |  |  |  |  |  |  | -4.96(-5.59,-4.32) |  | 1.46 | 0.226 |

^a^ $Logit\left(Y=Case_{\left\{0\left|1\right.\right\}}\right)=\beta X_{\text{RΙ-1}}+\delta$, and $Logit\left(Y=Case_{\left\{0\left|1\right.\right\}}\right)=\beta X_{\text{R}II\text{-1}}+\delta$

^b^ Heterogeneity calculated by formula $Q=\sum_{i} \left\{\left(Var_{i}\right)^{-1}\times\left(\beta_{i}-\beta_{pooled}\right)^{2}\right\}$, where $Var_{i}=\left\{\left(upper\lim it-lower\lim it\right)\div\left(2\times Z\right)\right\}^{2}$

**Table S4.** Linear regression analyses of the genetic associations among SNPs and lung function, smoking amount in controls.

|  | Controls ( N = 999 ) | | | | | | | | | | | | | | |
| --- | --- | --- | --- | --- | --- | --- | --- | --- | --- | --- | --- | --- | --- | --- | --- |
|  | FEV1 | |  | FEV1 pre-BD | |  | FEV1/FVC | |  |  | Pack-years | |  | Smoking Status | |
|  | β(SE) | *P*^a^ |  | β(SE) | *P*^a^ |  | β(SE) | *P*^a^ |  |  | β(SE) | *P*^a^ |  | β(SE) | *P*^a^ |
| rs12509311 | -0.024(0.025) | 0.346 |  | -0.012(0.019) | 0.533 |  | 0.224(0.336) | 0.504 |  |  | 1.346(0.871) | 0.123 |  | -0.032(0.044) | 0.475 |
| rs13118928 | -0.019(0.026) | 0.460 |  | -0.013(0.019) | 0.502 |  | 0.286(0.333) | 0.391 |  |  | 1.272(0.866) | 0.142 |  | -0.041(0.044) | 0.353 |
| rs1828591 | -0.024(0.025) | 0.346 |  | -0.012(0.019) | 0.533 |  | 0.224(0.336) | 0.504 |  |  | 1.346(0.871) | 0.123 |  | -0.032(0.044) | 0.475 |

^a^ calculated with adjusting gender and pack-years of smoking in linear regression;

The data presented are β (SE) with two-sided *P*-value; FEV1, forced expiratory volume in 1 s; FVC, forced vital capacity;

In Bold is *P* < 0.05.

**Table S5.** Interaction effects between SNPs and pack-year or age.

| Dependent variables | |  | Independent |  | Interaction effect | | |
| --- | --- | --- | --- | --- | --- | --- | --- |
|  |  |  | variables |  | Model | β(SE) | *P*^a^ |
| Group{0=control,1=copd} | |  |  |  |  |  |  |
|  |  |  | SNP×Py |  | rs12509311 × Py | -0.007(0.002) | 0.763 |
|  |  |  |  |  | rs13118928 × Py | -0.007(0.002) | 0.802 |
|  |  |  |  |  | rs1828591 × Py | -0.007(0.002) | 0.763 |
|  |  |  | SNP×Age |  | rs12509311 × Age | -0.014(0.011) | 0.231 |
|  |  |  |  |  | rs13118928 × Age | -0.014(0.011) | 0.234 |
|  |  |  |  |  | rs1828591 × Age | -0.014(0.011) | 0.231 |
| Lung fuction in COPD | |  |  |  |  |  |  |
|  | FEV1 |  | SNP×Py |  | rs12509311 × Py | -0.002(0.001) | 0.099 |
|  |  |  |  |  | rs13118928 × Py | -0.002(0.001) | 0.095 |
|  |  |  |  |  | rs1828591 × Py | -0.002(0.001) | 0.099 |
|  |  |  | SNP×Age |  | rs12509311 × Age | 0.002(0.001) | 0.667 |
|  |  |  |  |  | rs13118928 × Age | 0.002(0.001) | 0.666 |
|  |  |  |  |  | rs1828591 × Age | 0.002(0.001) | 0.667 |
|  | FEV1/FVC |  | SNP×Py |  | rs12509311 × Py | -0.071(0.034) | **0.037** |
|  |  |  |  |  | rs13118928 × Py | -0.071(0.034) | **0.035** |
|  |  |  |  |  | rs1828591 × Py | -0.071(0.034) | **0.037** |
|  |  |  | SNP×Age |  | rs12509311 × Age | 0.074(0.051) | 0.493 |
|  |  |  |  |  | rs13118928 × Age | 0.074(0.051) | 0.493 |
|  |  |  |  |  | rs1828591 × Age | 0.074(0.051) | 0.493 |
|  | FEV1 pre-BD |  | SNP×Py |  | rs12509311 × Py | 0.001(0.001) | 0.630 |
|  |  |  |  |  | rs13118928 × Py | 0.001(0.001) | 0.633 |
|  |  |  |  |  | rs1828591 × Py | 0.001(0.001) | 0.630 |
|  |  |  | SNP×Age |  | rs12509311 × Age | 0.002(0.001) | 0.216 |
|  |  |  |  |  | rs13118928 × Age | 0.002(0.001) | 0.216 |
|  |  |  |  |  | rs1828591 × Age | 0.002(0.001) | 0.216 |

^a^ calculated with adjusting gender and pack-years of smoking in linear or logistic regression;

The data presented are β (SE) with two-sided *P*-value; FEV1, forced expiratory volume in 1 s; FVC, forced vital capacity; Py, pack-year of smoking;

In Bold is *P* < 0.05.
